# Advances in Single-Cell Printing

**DOI:** 10.3390/mi13010080

**Published:** 2022-01-03

**Authors:** Xiaohu Zhou, Han Wu, Haotian Wen, Bo Zheng

**Affiliations:** Shenzhen Bay Laboratory, Institute of Cell Analysis, Shenzhen 518132, China; zhouxh@szbl.ac.cn (X.Z.); wuhan@szbl.ac.cn (H.W.); wenht@szbl.ac.cn (H.W.)

**Keywords:** inkjet printing, cell array, single-cell analysis, screening, microfluidics

## Abstract

Single-cell analysis is becoming an indispensable tool in modern biological and medical research. Single-cell isolation is the key step for single-cell analysis. Single-cell printing shows several distinct advantages among the single-cell isolation techniques, such as precise deposition, high encapsulation efficiency, and easy recovery. Therefore, recent developments in single-cell printing have attracted extensive attention. We review herein the recently developed bioprinting strategies with single-cell resolution, with a special focus on inkjet-like single-cell printing. First, we discuss the common cell printing strategies and introduce several typical and advanced printing strategies. Then, we introduce several typical applications based on single-cell printing, from single-cell array screening and mass spectrometry-based single-cell analysis to three-dimensional tissue formation. In the last part, we discuss the pros and cons of the single-cell strategies and provide a brief outlook for single-cell printing.

## 1. Introduction

Cells are the basic building blocks and functional units of all living systems. Exploring the component, structures, functions, and activity of cells could reveal the fundamental rules underpinning living systems. Most of our current knowledge on living systems is based on studies at the population level. However, due to the universal cell heterogeneity across all individual cells, population-level studies cannot provide detailed and accurate enough information for a deep understanding of the essential and fundamental laws of biological systems [1,2]. Therefore, directly exploring cellular structure and function at the single-cell level is becoming an indispensable tool to further reveal the essential secret of living systems. In the past decade, a plethora of techniques has been developed for single-cell analysis in various biomedical fields [2,3], including single-cell omics [4,5,6,7,8], single-cell biomechanical properties [9,10,11], cell–cell interactions [12,13,14], cell differentiation [15,16,17], cell subpopulation identification [18,19,20], cancer mechanisms [21,22,23], immunology [24,25], and neurology [26,27,28].

As the key step for single-cell analysis, single-cell isolation has attracted great attention, and a number of techniques have been developed for single-cell isolation and manipulation [29]. Limiting dilution [30] and fluorescence-activated cell sorting (FACS) [31] are two widely used techniques for single-cell isolation [32]. However, the low efficiency of the limiting dilution and the special equipment as well as the professional experience required for FACS limit their applications [29,32]. The development of single-cell printing has helped alleviate the situation, which utilizes various microfluidic technologies for single-cell isolation and analysis, such as droplet microfluidics [6,33,34], microwell arrays [35,36], and hydrodynamic traps [37,38]. Single-cell printing has several distinct advantages. First, single-cell printing can effectively and precisely deposit cells at specific sites in high throughput [39]. Second, the printed single cells or colonies can be easily recovered with addressability for subsequent analysis. Third, it is convenient to integrate the highly efficient single-cell printing with other techniques, such as imaging system [40,41], electric field [42,43,44], and acoustic field [45,46], and the single-cell encapsulation efficiency can reach more than 90%. By comparison, the theoretical limit of the single-cell capture efficiency of the widely used droplet-based microfluidic approach is only 37% according to Poisson’s distribution. Furthermore, bioprinting with the single-cell resolution can not only print 2D single-cell arrays for single-cell analysis but also three-dimensional tissue matrixes and organs for tissue engineering, drug discovery, and toxicology [47,48].

In this brief review, we summarize the recently developed bioprinting strategies with single-cell resolution, with a special focus on inkjet-like single-cell printing (Table S1 in Supplementary File). First, the common cell printing strategies, from contact printing to noncontact printing, are discussed. Several typical and advanced printing strategies are introduced. Then, some typical applications with various single-cell printing strategies are introduced, from single-cell array screening, mass spectrometry-based single-cell analysis to three-dimensional tissue formation. In the end, we summarize the pros and cons of single-cell printing strategies and provide a brief outlook on single-cell printing technology.

## 2. Printing Strategies

### 2.1. Contact Printing

In the early days, microcontact printing was first introduced to print the cell patterns at single-cell resolution with the development of soft lithography [49,50,51]. Microcontact printing is a technique that deposits ink materials of interest from an elastomeric stamp, typically polydimethylsiloxane (PDMS), with the desired motif onto a substrate through contact transfer [52]. The resulting material’s pattern on the substrate follows the topographic patterns of the stamp, similarly to the stamping process. The ink materials applied in microcontact printing vary from small molecules [53,54] and polymers [55] to biomolecules such as oligonucleotides [56] and proteins [57,58]. Theoretically, cells can also be printed directly onto substrates when using the cell solution as the ink. However, cell viability could be affected in practice when stress is exercised on the cell during the transferring process. To obtain cell arrays with microcontact printing, it is more common to print cell adherent molecules onto the substrate through microcontact printing first and then apply cells on the substrate to form the cell array [59,60,61,62,63].

In contact printing of cells, cell adherent molecules such as extracellular matrix (ECM) proteins including fibronectin, laminin, and vitronectin are generally used [63,64]. These functional anchoring units are usually immobilized on surface-active molecules, such as self-assemble monolayers (SAMs) or other functional groups, which were previously printed on substrates with microcontact printing [65]. The cell adherent molecules take the pattern of the surface-active molecules determined by the stamp, and eventually, the cells will take the shape of the printed patterns. Hence, the size of the printed patterns on the substrate is of great importance to achieve a single-cell resolution printing. The area of the printed adhesive pattern is supposed to be identical to the cells’ spreading area, resulting in a demand for a high-resolution printing method.

**Figure 1:**
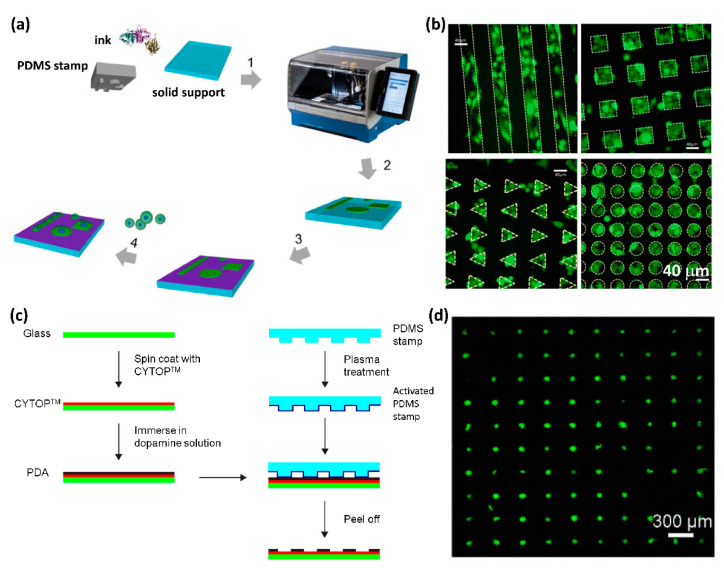
(**a**) Overall microcontact printing of adhesive patterns using InnoStamp 40. (**b**) Fluorescence images of PC3-GFP cells immobilized on fibronectin micropatterns of various shapes. Patterns are depicted in dashed lines. Scale bar: 40 µm. Reproduced with permission from [59]. (**c**) Schematic illustration of the fabrication of polydopamine patterns on CYTOP-coated glass surface by negative microcontact printing. (**d**) Confocal image of the patterned single mouse mesenchymal stem cell array on polydopamine patterned CYTOP surface. Reproduced with permission from [60].

Based on microcontact printing, polymer pen lithography, which can generate patterns with spot sizes ranging from 90 nm to hundreds of µm, was described by Mirkin’s group [66], allowing the creation of high-resolution micropatterns for single-cell patterning. Furthermore, other approaches for single-cell printing with high throughput and efficiency have also been developed recently based on microcontact printing. Foncy et al. proposed an automated microcontact printing method to produce a biomolecule microarray composed of extracellular matrix protein for the preparation of cell microarrays (Figure 1a,b) [56]. In the process, a microcontact printer, which can handle the PDMS stamp and control the stamping process with a magnetic field, was employed, resulting in the uniform printing of defined biomolecular patterns. With the printer, the stamping process was effective, robust, and repeatable, allowing for the generation of large-scale cell microarrays. We also developed a negative microcontact printing method with sub-micrometer pattern resolution for single-cell patterning (Figure 1c,d) [60,67]. Unlike the conventional microcontact printing, which prints molecules onto substrates, in the negative microcontact printing, the ink molecules, consisting of polydopamine (PDA), were previously coated on a hydrophobic substrate and were removed at the contact area by the PDMS stamp, leaving behind a complementary pattern to the stamp on the surface. With the hydrophilic–hydrophobic microarray created by negative microcontact printing, we further generated a single-cell array of mouse mesenchymal stem cells with an efficiency of 94%, i.e., 94% of PDA spots on the substrate were occupied with single cells by exploiting different cell adhesion behaviors on the hydrophilic and hydrophobic surfaces.

However, there are several intricate drawbacks of the contact printing strategy. First, complex microfabrication and surface modification are required for stamp preparation. Second, large-scale production of the single-cell array with good uniformity and reproducibility relies on automated stamping controlling system, as the manually controlled stamping process lacks uniform and precise stress control over stamps during contact printing and severely impairs the pattern quality. Third, the risk for cross-contamination is relatively high during stamping. Finally, contact printing is only suitable for 2D cell array construction but not for the complex 3D multicellular structure formation.

### 2.2. Noncontact Printing

To overcome the problems of contact printing, various noncontact printing strategies have been developed to directly print cell patterns, such as extrusion printing, laser-based cell printing, and inkjet bioprinting [47,68]. Extrusion-based cell printing involves the extrusion of cell-laden inks through nozzles with either mechanical or pneumatic forces [47,69]. Extrusion-based cell printing has been widely applied to fabricating the three-dimensional tissues and organs with the capability of multiple cell types and materials to create controlled cell heterogeneity in the printing product [47,69,70]. However, extrusion-based printing generally dispenses the cell-laden inks as fibers with a relatively low resolution of ~100 μm, which makes the method unsuitable for single-cell printing [47]. Contrarily, laser-based cell printing has long been utilized in single-cell printing because laser printing can precisely select and position live cells on a predefined location from a cell suspension with high density [71,72,73,74,75]. In laser-based cell printing, cells are pre-loaded on a laser-sensitive surface, and then the laser is applied to transfer the target cells onto an underlying substrate [47]. However, the time requirement and limited throughput are the major drawbacks of laser-based single-cell printing. Furthermore, the concern of the laser radiation-induced damage on the printed cells, expensive printer components, and complicated setup and operation limit the applications of laser-based cell printing [74,75,76].

Compared to other printing strategies, inkjet and inkjet-like bioprinting with the drop-on-demand mode show several distinct advantages, which make them the most promising strategy to print products ranging from single-cell arrays to three-dimensional tissues and organs. Drop-on-demand inkjet-like cell printing usually jets out the cell-laden inks as droplets through micro-nozzles onto a substrate upon actuation [68]. The advantages are multifold. First, inkjet-like printing can achieve the single-cell resolution with each drop containing a single cell. Second, inkjet-like printing shows a smaller printing footprint, which minimizes the interference to the subsequent analysis. Third, inkjet-like printing is easily scalable for high throughput production with multiple printheads. Finally, inkjet-like printing is environmentally friendly with less raw material waste [47,68,74,77,78,79].

In the early days, the consumer paper inkjet printers were usually adapted for cell printing, including both the traditional thermal printers and the piezoelectric printers [39,68,80]. Boland and co-workers pioneered the inkjet printing technology to print mammalian cells suspended in a cell culture medium with the modified commercial paper inkjet printer in 2003 [81,82]. However, the applications of these bioprinters were limited by several issues. For example, special bio-inks were usually required for cell suspensions to maintain cell viability from the high mechanical pressure or high temperature. The ejection nozzle was also frequently clogged by cells. The encapsulation of cells was unreliable. Furthermore, single-cell bioprinting with the inkjet printing method could be achieved only by diluting the cell suspension to reduce the cell density [80,82,83]. Therefore, to increase the reliability and efficiency of single-cell printing, various new techniques have been developed to improve the dispensing efficiency of inkjet-like single-cell printing [40,41,42,44,45,46,68,84,85,86,87,88,89,90,91,92,93,94,95,96,97,98,99].

#### 2.2.1. Acoustic Field-Based Single-Cell Printing

**Figure 2:**
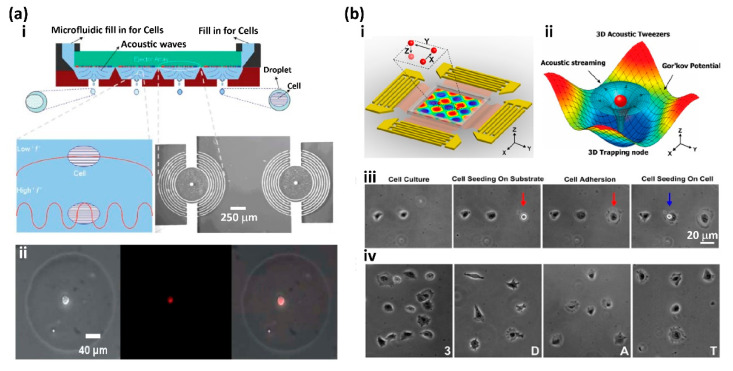
Acoustic field-assisted single-cell printing: (**a**-**i**) The setup of the acoustic picolitre droplet generator. (**a**-**ii**) A single 3T3 fibroblast cell with cell tracker dye was printed on a glass substrate (from left to right: white field image, fluorescent image, and the overlap image). Reproduced with permission from [45]. (**b**-**i**) Schematic illustration of the planar surface acoustic wave generators. (**b**-**ii**) Numerical simulation results of the surface acoustic wave-based 3D acoustic tweezer. (**b**-**iii**) The single 3T3 mouse fibroblast can be precisely printed to the desired position, either on the substrate to form a linear cell array (red arrow) or on the top of another cell (blue arrow). (**b**-**iv**) HeLa S3 cells were printed on a substrate to form the pattern of letters: “3” “D” “A” “T”. Reproduced with permission from [46].

Gentle acoustic fields with the ability to maintain cells in their native state in their original culture without harm shows the potential to generate and print the droplet containing single cells [45,46,100,101,102,103,104,105]. In 2007, Demirci et al. applied a gentle acoustic field to generate picolitre droplets containing cells from a microfluidic chip and increased the reliability of cell encapsulation efficiency by as high as 98.4% (Figure 2a) [45]. In the process, interdigital gold rings were placed on a surface acoustic wave piezoelectric substrate, and a sinusoidal electrical signal at the resonance frequency of the device was applied to generate the surface acoustic waves on demand. These generated acoustic waves propagate through the cell suspension for droplet generation. With the circular geometry of the interdigital gold rings, the acoustic focusing point forms at the interface between the air and the cell suspension under each gold ring, forming an acoustic droplet ejector, which ejects picolitre droplets with the drop-on-demand mode. With this design, an array of droplet ejectors can be easily integrated for high throughput droplet generation. This acoustic printer can generate droplets of uniform size with diameters from 2 μm to 200 μm. To prevent acoustic waves from harming the cells, the wavelength of the acoustic wave was chosen to be larger than the size of the cells (Figure 2(a-i)). Five different kinds of cells were applied to demonstrate the capability of the acoustic printer for live-cell printing, including mouse embryonic stem cells (mESCs), 3T3 fibroblasts, AML-12 hepatocytes, Raji cells, and HL-1 cardiomyocytes. These cells were encapsulated in acoustic picolitre droplets with a diameter of about 37 μm and ejected at a rate from 1 to 10,000 drops per second. The overall cell viability of these cells after printing reached 89.8%. However, although this acoustic cell printer can increase the reliability of cell encapsulation from the ~60% rate of the inkjet printer to 98.4%, it cannot guarantee that each acoustic picolitre droplet only contained a single cell [45].

Then, Huang and co-workers developed a three-dimensional acoustic tweezer based on the surface acoustic wave, which can precisely pick up, translate, and print single cells (Figure 2b) [46]. As shown in Figure 2(b-i), two mutually orthogonal pairs of interdigital transducers were superimposed on a lithium niobate piezoelectric substrate for the generation of surface acoustic waves. A double-channel radio-frequency signal generator and two amplifiers were individually connected to each pair of interdigital transducers to generate surface acoustic waves with different frequencies. The surface acoustic waves propagate through the microfluidic chamber to produce a three-dimensional acoustic field and induce the three-dimensional acoustic streaming to form stable 3D trapping nodes, and the reflected acoustic waves by the microfluidic chamber wall form a Gor’kov potential field (Figure 2(b-ii)). These 3D trapping nodes can be precisely transported in a horizontal plane by tuning the phase angle of the interdigital transducer pairs and manipulated vertically by tuning the input power of the acoustic field. Therefore, living cells can be precisely printed onto the desired location with this 3D acoustic tweezer when the cells are captured by the 3D trapping nodes. To demonstrate this acoustic tweezer-based single-cell printing, a single 3T3 mouse fibroblast was captured and printed onto the desired position on the substrate to form a linear cell array, and the position of the printed cell was precisely controlled (Figure 2(b-iii)). HeLa S3 cells were also printed on a substrate to form the pattern of letters: “3” “D” “A” “T” (Figure 2(b-iv)) [46].

#### 2.2.2. Label-Free Computer Vision-Based Single-Cell Printing

Besides the acoustic field-based inkjet-like single-cell printing, the other popular and useful strategy used to increase the reliability and efficiency of single-cell printing is integrating real-time monitoring techniques with a microfluidic dispenser or printer [32,40,41,42,97,98,106]. These monitoring techniques can detect and monitor the cells to be printed and trigger the integrated microfluidic dispenser or printer to generate and print droplets containing single cells.

**Figure 3:**
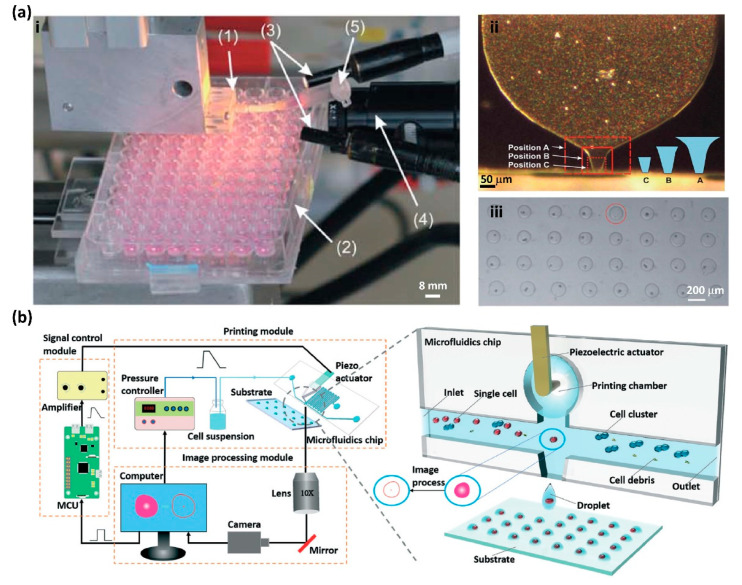
(**a-i**) The single-cell printer consists of a dispenser chip (1) mounted to an aluminum case with a piezo stack actuator, a substrate, or microplate (2) for single-cell printing, an illumination system (3), a CCD camera (4) for cell detection, and a reservoir (5) for cell suspension loading. (**a**-**ii**) The size of the ROI was determined by the volume of the printed droplet. (**a**-**iii**) A single HeLa cell array was printed on a substrate. Reproduced with permission from [40]. (**b**) Schematic illustration of the real-time cellular recognition-based single-cell printing system using the microfluidic dispenser chip and droplet generation. Reproduced with permission from [41].

Koltay and co-workers developed an inkjet printing-based single-cell manipulator (SCM) by integrating an inkjet-like printing system with a computer vision system (Figure 3a) [40]. The SCM can efficiently encapsulate a single cell in a picolitre droplet from a cell suspension and then print these droplets with single cells on defined positions of the substrate. The SCM consists of a dispenser chip, an optical detection system, a control algorithm, and a motion control system (Figure 3(a-i)). The dispenser chip was made from silicon and glass, which makes it transparent for optical view. A piezo stack actuator is used to generate the free-flying droplet through the dispenser chip. A digital camera is used to monitor the status of the cell distribution inside the region of interest (ROI) at the nozzle section of the dispenser chip. The number of cells in the subsequently printed droplet is predicted by an algorithm based on the status of cell distribution in the ROI. The size of the ROI is determined by the volume of the printed droplet (Figure 3(a-ii)). The subsequent droplet would be printed on the target position only if there was a single cell in the ROI. If there is zero or more than one cell in the ROI, the next printed droplet is delivered to the waste position. Therefore, the computer vision system can guarantee that only droplets containing a single cell are delivered to the target position, achieving high-efficiency single-cell printing (Figure 3(a-iii)). This SCM system was applied to print HeLa cells, and the printing efficiency reaches as high as 87%, and the cell viability rate after printing can be as high as 75% [40]. Then, to further increase the efficiency of single-cell printing, a controllable micro-pneumatic shutter system was installed below the nozzle of the dispenser chip to remove droplets with either zero or multiple cells [81]. Recently, Riba et al. combined this single-cell printer with a machine learning-based image classification for real-time cell viability sorting [98]. An extremely shallow convolution neural network was successfully applied for the classification of cell images with low computational effort, which made it suitable for real-time classification. With this machine learning-based classifier, the clone recovery of the CHO-K1 cells with a large fraction of dead cells can be increased from 27% to 73% [98].

Recently, Chu and co-workers developed another computer vision-based highly efficient single-cell printer by integrating real-time cell recognition and microfluidic impact printing (Figure 3b) [41]. This single-cell printer consists of a printing module, a signal control module, and an imaging processing module (Figure 3b). The pressure controller is applied to dispense cell suspension into the microchip. A high-speed camera is used to capture images of the detection zone at the crossing, which are processed in real-time to identify the status of the cell distribution at the detection zone. If the target single cell is identified, a piezoelectric actuator is triggered to strike a flexible membrane on the printer chamber to generate a droplet within the target single cell to the defined location. In the demonstration, this single-cell printer printed the HeLa cell with an efficiency of 90.3% at a throughput of 2 Hz, and the cell viability reached 96.6% after printing [41].

#### 2.2.3. Other Methods

**Figure 4:**
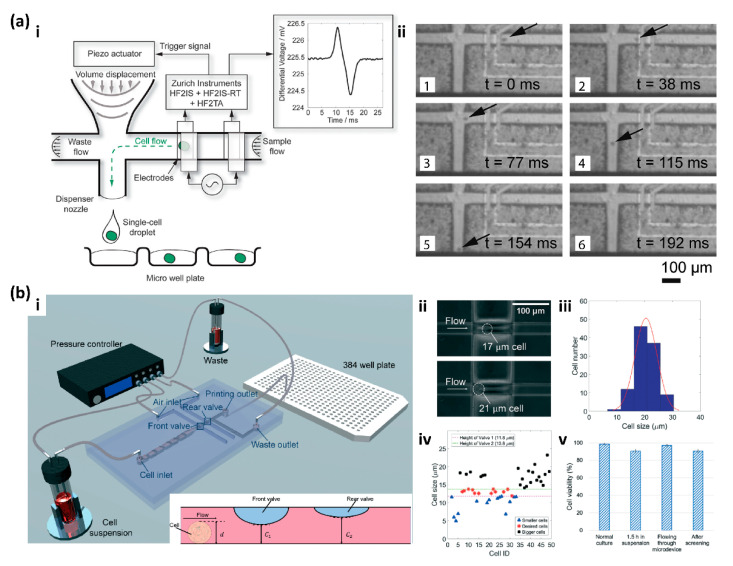
(**a-i**) Schematic illustration of the mechanism of the impedance-based single-cell printing system. The impedance signals of the flow-through cells trigger the actuator to generate and dispense the droplets containing single cells. (**a-ii**) The time-lapse images show the progress of printing a single HeLa cell. Reproduced with permission from [42]. (**b-i**) Schematic illustration of the system and mechanism of the dual microvalves-based single-cell screening and printing. (**b**-**ii**) HUVECs were captured by the valves under the pressure of 0.8 atm. (**b**-**iii**) the size distribution of the HUVECs’ suspension. (**b**-**iv**) the size of HUVECs screened by the valves. (**b**-**v**) Cell viability with different conditions. Reproduced with permission from [99].

Besides utilizing the computer vision system for real-time cell detecting, researchers also developed other techniques to increase the printing efficiency of inkjet-type single-cell printing. Feng et al. integrated a pair of capacitance sensors in a microfluidic air ejector to detect the oocyte cells and achieved accurate single-oocyte printing with the assistance of the micropillars formed semicircular bay [43]. However, only extra-large cells such as oocytes with a diameter of about 100 μm can be printed by the printer. Schoendube et al. introduced another electric excitation-based cell detection system to trigger single-cell printing by integrating the impedance flow cytometry in a microfluidics dispenser chip (Figure 4a) [42]. When the cells flow through the microfluidic chip, the integrated electrodes measure the channel impedance. The differential signals are used to reduce undesired perturbations. The flow-through cell generates a positive peak at the first electrode pair and a negative signal at the second electrode pair. Once the signal of the flow-through cell is detected, a piezoelectric actuator is triggered to deflect a silicone membrane to generate and dispense a free-flying droplet containing the single cell (Figure 4(a-i)). HeLa cells were used to demonstrate the capability of the printer for single-cell printing (Figure 4(a-ii)) [42].

Size-based cell screening and separation strategies have been widely used in microfluidic platforms. Chen and co-workers introduced a microfluidic printer with dual microvalves that can dynamically screen and print single cells (Figure 4b) [99]. This microfluidic printer consists of three layers: a gas layer to control the two pneumatic valves, a membrane layer to separate the gas and fluid, and a fluid layer with a flow channel for cell suspension. When the cells flow through the microchannel, the front and rear valves are independently activated to control the cells with the desired size for printing (Figure 4(b-i)). When the cell on demand is selected by the valves, positive pressure is applied from the waste outlet to generate the printing of the droplet containing the selected cell into the microplate; otherwise, negative pressure is applied to collect the waste. HUVECs were utilized to demonstrate the capability of this printer for screening and printing single cells with the desired size range. When a pressure of 0.8 atm was applied to the valve, the 17 μm and 21 μm cells were captured (Figure 4(b-ii)). The HUVECs’ suspension with the size ranging from 10 to 30 μm (Figure 4(b-iii)) was dispensed into the microfluidic chip for screening and printing, and only the cells with the desired size were selected (Figure 4(b-iv)). The efficiency of this microfluidic printer for printing single cells was 100%, and cell viability after printing reached 90.6% (Figure 4(b-v)) [99].

## 3. Applications

As mentioned previously, single-cell analysis has become a powerful and indispensable tool in modern biological and medical research [2,3]. With the development of single-cell printing in the past decade, various single-cell printing-based single-cell analyses and applications have been performed, ranging from single-cell array-based screening [89,90,93,107,108,109] and single-cell based mass spectroscopy [110,111,112,113,114,115] to live three-dimensional tissue formation [47,48,116].

### 3.1. High Throughput Screening

**Figure 5:**
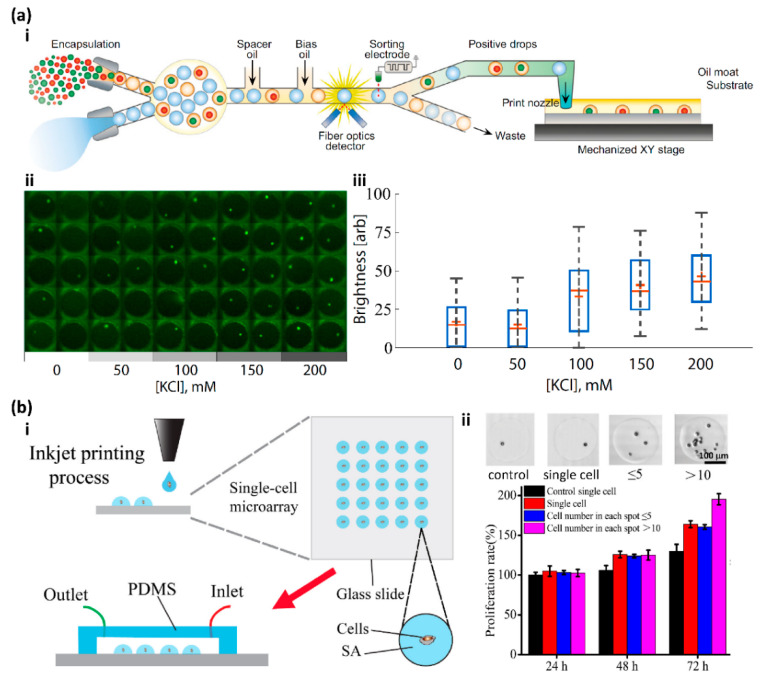
(**a**-**i**) Schematic diagram of the fluorescence-activated cell sorter-based printed droplet microfluidics. (**a**-**ii**) The intracellular calcium release assay was screened on a single PC3 prostate cancer cell array with different concentrations of KCl. (**a**-**iii**) Box plots show the results of the single-cell-based intracellular calcium release assay. Reproduced with permission from [93]. (**b**-**i**) Schematic diagram of the inkjet printing process to fabricate the single-cell microarray. (**b**-**ii**) ATP-induced proliferation experiment indicated that the multi-cell group had a higher proliferation rate than the single cell. Reproduced with permission from [89].

Recently, Cole et al. developed printed droplet microfluidics (PDM) to print droplets containing single cells and reagents with deterministic control by integrating the fluorescence-activated droplet sorter, which provides the capability of selecting the droplets containing the desired cells and reagents from a set of candidates and printing them on a motorized substrate (Figure 5(a-i)) [93]. To demonstrate the ability of the PDM for single-cell analysis, a time-sensitive single-cell calcium release assay was performed. PC3 prostate cancer cells with a green-fluorescing Ca^2+^ indicator dye were printed as a single-cell array (Figure 5(a-ii)). KCl was used to depolarize the cell membrane to induce intracellular calcium release. The results show that the higher concentration of KCl induced more detectable Ca^2+^ signals, which was consistent with the bulk experiments (Figure 5(a-iii)) [93].

More recently, Zhou and co-workers developed a laboratory-made inkjet printing system to construct single-cell arrays (Figure 5(b-i)) [89]. This modified inkjet printer can precisely control the number of cells in each printing spot on a hydrophobic substrate for subsequent in-depth research, and the single-cell occupancy reaches as high as 91%. Single-cell arrays of MCF-7 cells with a DMEM medium and sodium alginate were constructed with this modified inkjet printing system, and the real-time single-cell assays showed high activity and proliferation, low levels of ROS, and cell apoptosis, which demonstrated the capability of this inkjet printing system for single-cell study. Interestingly, in the ATP-induced proliferation experiment, they found that extracellular ATP can indeed significantly increase MCF-7 cell proliferation over 72 h as reported, and the multi-cell group showed a higher proliferation rate than the single-cell group, which indicates that cell communication might also play an important role in cell proliferation (Figure 5(b-ii)) [89].

### 3.2. Mass Spectrometry Based Single-Cell Analysis

Mass spectrometry (MS) is a powerful tool to qualitatively and quantitatively detect molecules at the femtomolar sensitivity without a labeling requirement [117]. Furthermore, the multiplex detection with high throughput and low sample consumption makes MS the ideal tool for single-cell analysis [110,111,112,113,114,115], especially for single-cell proteomics [8,118,119].

**Figure 6:**
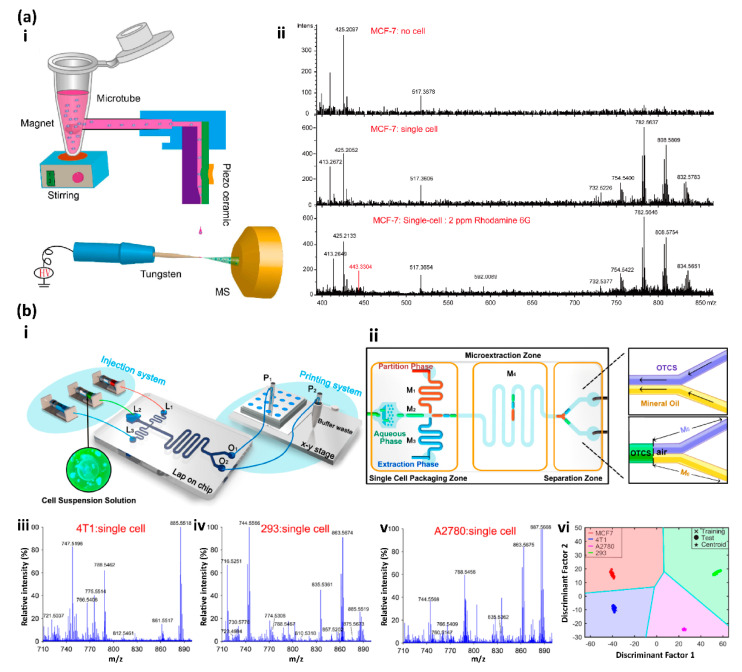
(**a-i**) Schematic illustration of the experiment setup: (**a**-**ii**) Detection of the single MCF cell labeled with Rhodamine 6G. Reproduced with permission from [113]. (**b-i**) Schematic illustration of the three-phase single-cell printing (TP-SCP) system. (**b**-**ii**) Schematic diagram of the microfluidic chip with the three functional zones. (**b-iii–b-v**) MS spectra of (**b**-**iii**) 4T1 single cells, (**b**-**iv**) 293 single cells and (**b**-**v**) A2780 single cells. (**b**-**vi**) The classification result of the four types of cells. Reproduced with permission from [110].

Recently, Lin and co-workers developed an MS-based single-cell analysis strategy by integrating the drop-on-demand inkjet printing with the probe electrospray ionization (ESI) mass spectrometry (Figure 6(a-i)) [113]. The free-flying droplets containing single cells were generated from a homemade piezoelectric inkjet printer and precisely printed onto the tungsten probe tip of the ESI needle [115]. The high voltage applied on the needle would immediately spray and ionize the droplets to the MS detector. To increase the single-cell-droplet percentage, the cell suspension was stirred on a homemade magnetic stirring machine to maintain the homogeneous distribution during printing, which increased the single-cell-droplet percentage from 37% at the random dispersion to 43.8%. Since lipids are often involved in many vital cell physiological processes [120], single-cell cellular surface phospholipids profiling was performed to demonstrate the capability of this system for single-cell analysis. Eight different types of single cells were successfully screened and differentiated by their lipid fingerprints, which were obtained with this system. Furthermore, this system differentiated the single Rhodamine 6G labeled single MCF-7 cell from unlabeled cells (Figure 6(a-ii)), which indicates the capability of cell marker detection [113].

Recently, Zhang and co-workers developed a three-phase droplet-based single-cell printing analysis system (TP-SCP) by combining a microfluidic chip with matrix-assisted laser desorption/ionization mass spectrometry, which eliminates the matrix effect and directly analyzes live single cells in their native state (Figure 6(b-i)) [110]. The microfluidic chip of the TP-SCP system has three zones: the single-cell package zone where the droplets containing the single cells in PBS buffer were generated, accompanied by the droplets containing the extraction phase and droplets with partition phase; the microextraction zone where the water-soluble substance in cells was extracted; the separation zone where the extraction phase of the single cells and aqueous phase of cell residual liquid were separated for subsequent MS analysis and collection (Figure 6(b-ii)). To achieve the phase separation, the microchannel M5 was modified to be hydrophobic, while the microchannel M6 was hydrophilic. Cell classification was performed to test the performance of the TP-SCP system for single-cell analysis. The partial main phospholipids of four types of cells (MCF-7, 4T1, 293, and A2780) were profiled at the single-cell resolution with the TP-SCP system (Figure 6(b-iii–b-v)), and both the principal component analysis and linear discriminant analysis algorithms were used to successfully classify the four types of cells with an accuracy rate of 100% (Figure 6(b-vi)) [110].

### 3.3. 3D Tissue Printing

Printing three-dimensional functional live tissues or organs is one of the most important applications of cell bioprinting, not only for academic research and industrial development but also for clinical practice [39,47,48,68,69,74,79,121]. However, current bioprinters for the 3D bioprinting of tissues and organs cannot print live functional tissues and organs with single-cell resolution, which is required for real functional tissues and organs [47,48,79,116].

**Figure 7:**
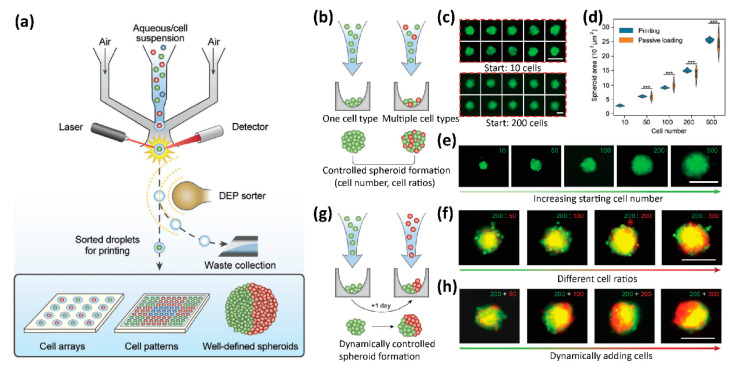
(**a**) Schematic illustration of the high-definition single-cell printing (HD-SCP) system. (**b**) Schematic illustration of controlled spheroid formation with HD-SCP. (**c**–**e**) The size of the spheroids can be precisely controlled by the initial number of the printed single cells. (**f**) Multicellular spheroids formed by printing multiple cell types with different cell ratios. (**g**) Schematic illustration of dynamically controlled spheroid formation with HS-SCP. (**h**) Bioprinting multicellular Janus spheroids. Scale bars: 100 μm for (**c**) upper, and 200 μm for (**c**) lower, (**e**,**f**,**h**). Reproduced with permission from [116].

Recently, Abate and co-workers developed a high-definition single-cell printing system (HD-SCP), which can reliably print the single cells of interest from a bunch of multiple candidates with high accuracy and speed (Figure 7) [116]. The HD-SCP system integrated a miniaturized FACS-based cell sorter in a microfluidic air ejector (Figure 7a). Cells to be printed were labeled with fluorescence dyes for sorting. The miniaturized cell sorter has two functional zones: the fluorescent detection zone to identify the desired single cells and the dielectrophoresis-based sorting zone to sort droplets by deflecting the undesired droplets to the downstream vacuum channel. When the sprayed droplets passed through the detection zone, the fluorescence signals were detected and analyzed in real-time by a four-color detector. Only the droplets containing the desired single cells are be printed to the predefined location, otherwise, the droplets are deflected by the dielectrophoresis sorter and collected by the vacuum channel as waste (Figure 7a). HD-SCP can print single-cell with the accuracy of 10 μm at the speed of about 100 Hz. To demonstrate the capability of HP-SCP for 3D bioprinting, the well-defined spheroids with controlled single cells and morphologies were printed (Figure 7b–h). The fine size of the spheroids can be precisely controlled by the initial number of the printed single cells (Figure 7c–e). Spheroids with two different cell compositions were also printed with HD-SCP (Figure 7b,f–h). When two different cells were printed sequentially at the same time (Figure 7b), the cells tended to aggregate together in the resultant spheroids (Figure 7f), whereas if the red cells were printed to the pre-formed spheroids, which had formed with only green cells for one day (Figure 7g), the multicellular Janus spheroids were formed (Figure 7h) [116].

## 4. Summary and Future Perspective

In this review, we have summarized the recently developed single-cell bioprinting strategies and highlighted several of the most important and recently developed strategies (Table S1). We also summarized several advanced single-cell bioprinting-based applications for single-cell analysis, including single-cell array screening, mass spectrometry-based single-cell analysis, and three-dimensional tissue formation.

Cell bioprinting has made remarkable progress in printing three-dimensional multicellular tissues and organs in the past decade [47,48,74]. However, compared to the 3D bioprinting, where the single-cell resolution is not necessarily required, there is little progress in single-cell printing-based single-cell analysis, although the recently developed single-cell bioprinting strategies show the great potential of single-cell analysis in-depth with promising advantages, such as high encapsulation efficiency, precise deposition, and easy recovery. Currently, the most common strategies for single-cell analysis (omics) are based on droplet microfluidics [33]. Several issues limit the applications of current single-cell printing for in-depth single-cell analysis. First and foremost, although the reliability and efficiency of the encapsulation of single cells are dramatically enhanced, the overall throughput of the single-cell printing is still low, at a rate of ~2 Hz [99]. Second, compared to the popular microfluidic technologies in an isolated system, current printing strategies normally rely on printing the single-cell droplets in an open environment, which may cause interference and deviation in the subsequent analysis. Third, many subsequent analyses after single-cell isolation are performed on instruments that are incompatible with single-cell printing [2,7].

Despite the above issues, single-cell printing is experiencing rapid development. Several commercial single-cell printers are now available. Throughput, single-cell efficiency, robustness, and reproducibility are among the most important factors to consider when evaluating a single-cell printing technique or product. With the increasing demand in tissue engineering, precision medicine, liquid biopsy, and drug discovery, we expect that single-cell printing will play a more vital role in single-cell analysis in the future.

## Data Availability

Not applicable.

## Supplementary Materials

The following are available online at https://www.mdpi.com/article/10.3390/mi13010080/s1, Table S1 Summary of the single-cell printing strategies.
