# Supplementary material for: Advances in Single-Cell Printing
Source: Micromachines (Basel). 2022 Jan 3;13(1):80. doi: 10.3390/mi13010080 (PMC8778191; doi:10.3390/mi13010080)
Supplement: Supplementary file 1 [file micromachines-13-00080-s001.zip › micromachines-1524906-supplementary.pdf]

Supporting Information

Advances in Single-Cell Printing

Xiaohu Zhou, Han Wu, Haotian Wen, Bo Zheng*

Institute of Cell Analysis, Shenzhen Bay Laboratory, Shenzhen 518132, Guangdong, China;
zhouxh@szbl.ac.cn (X.Z.); wuhan@szbl.ac.cn (H.W.); wenht@szbl.ac.cn (H.W.);
*Correspondence: bozheng@szbl.ac.cn

Table S1 Summary of the single-cell printing strategies

Technique	Mechanism	Performance	Application
**Contact printing**	Surface patterning by contract transfer	Micropattern size and shape controllable [56,57]<br>Cell immobilization selectivity: > 75% [56]<br>Single cell capture efficiency: 94% [57]	Cell patterns [56]<br>Single cell array [57]
**Acoustic field-assisted inkjet printing**	Acoustic field	Number of cells per printing: Random [45] or 1 [46]<br>Cell encapsulation efficiency: 98.4% [45]<br>Throughput: 1000 Hz [45]	Single cell array [45,46]<br>Cell patterns [46, 105]<br>Cell-laden 3D hydrogel structures [103]
**Label-free computer vision-based single-cell printing**	Piezoelectric actuation triggered by a computer vision system	Number of cells per printing: 1 or multiple [40, 41, 81, 98]<br>Single cell efficiency: 87% [40]; 90.3% [41]<br>Cell viability: 75% [40]; 96.6% [41]<br>Throughput: 2 Hz with 90.3% single-cell efficiency [41]	Single cell array [40,41, 81]<br>Single cell real-time sorting [98]
**Electric signals based single-cell printing**	Piezoelectric pressure actuated by an electric signal	Number of cells per printing: 1 or multiple [42]<br>Single-cell efficiency: 73% [42]	Single-cell array [42]
**Pneumatic valves-based single-cell printing**	Pneumatic pressure	Number of cells per printing: 1 [43, 99]<br>Single cell efficiency: ~100% [43, 99]<br>Cell viability: 70% [43]; 90.64% [99]	Single oocyte array [43]<br>Cell size screening [99]
**FACS based single-cell printing**	Dispensing droplets containing single cells based on the fluorescent signals	Number of cells per printing: 1 or multiple [93, 116]<br>Cell encapsulation efficiency: 99.5% [116]<br>Single cell efficiency: 98% [93]	Single cell arrays and cell patterns [93, 116]<br>Single-cell calcium release assay [93]

		Cell viability: 94% [116]<br>Throughput: 1.5 Hz [93]; 100 Hz [116]	3D well-defined spheroid [116]
**Limiting dilution based single-cell printing**	Dispensing droplets containing single cells with diluting cell suspension	Number of cells per printing: Random (almost Poisson distribution) [89, 110, 113]<br>Cell encapsulation efficiency: 46% [110]<br>Single cell efficiency: 91% (multiple droplets for each spot) [89]; 39% [110]; 43.8% [113]<br>Cell viability: 100% [89]<br>Throughput: 3~4 Hz [110]	Single cell assays [89]<br>Mass spectrometry-based single-cell analysis [110, 113]
